# Predicting patterns of failure in temporal lobe GBMs: possible implications on radiotherapy treatment portals

**DOI:** 10.1186/s13014-018-1078-y

**Published:** 2018-07-20

**Authors:** Dasantha Jayamanne, Helen Wheeler, David Brazier, Allison Newey, Marina Kastelan, Linxin Guo, Michael Back

**Affiliations:** 1 Northern Sydney Cancer Centre, Royal North Shore Hospital, Sydney, NSW Australia; 2 Northern Clinical School, Sydney Medical School, University of Sydney, Sydney, NSW Australia; 3 Department of Radiology, Royal North Shore Hospital, Sydney, NSW Australia; 4 Central Coast Cancer Centre, Gosford Hospital, Gosford, NSW Australia; 5 Sydney Neurooncology Group, Sydney, NSW Australia

**Keywords:** Glioblastoma, Radiation, Recurrence, Patterns of failure

## Abstract

**Background:**

Characterise patterns of failure of Temporal Lobe (TL) Glioblastoma (GBM) following treatment with relation to normal temporal lobe anatomy and neural pathways.

**Methods:**

335 GBM patients received radiotherapy between 03/2007 and 07/2014, 100 were located in TL. Site of initial tumour and subsequent relapse were subdivided into 5 local TL sites (anterior, lateral, medial, posterior and superior); 5 adjacent regional sites (occipital lobe, inferior frontal lobe, caudate/thalamus/internal/external capsules, fornix/ventricular trigone), and 5 distant failure sites (ventricles, contralateral hemisphere, brainstem, leptomeninges and spine). Extension along major neuroanatomical pathways at initial presentation and at first documented Magnetic Resonance Imaging (MRI) failure were categorised into anterior, superior, medial and posterior pathways.

**Results:**

Of the 100 patients, 86 had radiological progress with a median survival of 17.3 months.

At initial diagnosis, 74% of tumours were linked to one TL site and 94% were confined to the TL. 19% had neural pathway disease at initial pre-treatment MRI. At first recurrence locoregional site failure was 74%. 26% failed within distant sites and 53% patients were noted to have neural pathway involvement.

Initial tumour location predicted for local site recurrence (*p* < 0.0001), regional site recurrence (*p* = 0.004) and neural pathway recurrence pattern (*p* = 0.005), but not for distant sites (*p* = 0.081).

**Conclusion:**

Most GBMs fail at local or adjacent regional sites. Many of the recurrences occurred in a predictable pattern within a local or regional site, unique to initial TL site with more than half involving neural pathways. Knowledge of tumour infiltration and failure may improve target definition and radiotherapy.

## Background

Glioblastoma (GBM) is the most common primary malignant brain tumour and despite subtle advances in the past decade, survival remains poor beyond three years. Current standard of practice involves aggressive surgical resection followed by concurrent chemoradiotherapy followed by adjuvant chemotherapy [1]. Despite aggressive treatment, the prognosis for most patients is short and with a median survival of 14–18 months [2–4]. Several other therapeutic agents have been trialed in the adjuvant setting with no clear survival benefit [5, 6]. As such, there is a need to improve current treatment protocols. Glioblastoma tumor spread along white matter tract as found in the temporal lobe, could be one explanation of the high loco-regional recurrence rate of glioblastoma and thereby poor overall survival after standard therapy.

Radiotherapy has been shown to improve survival in malignant gliomas [7]. However, despite aggressive local treatment, with surgery and radiotherapy, 80% of gliomas fail within 2 cm of the resection cavity [8–11], Older studies evaluating hyperfractionated dose escalation show no improvement in tumour control or survival [12, 13]. Others have shown better control with accelerated very high doses of radiotherapy, up to 90Gy, but with unacceptable radiation necrosis [14]. Clearly, a greater understanding of tumour biology, patterns of failure and infiltration is needed to improve tumour control.

Glioma infiltration along white matter tracts has been proposed as a potential means of spread of tumour both locally and distantly within the brain [15]. Standard imaging techniques with computed tomography and magnetic resonance imaging (MRI) do not adequately delineate these regions. T2-weighted MRI Fluid Attenuated Inversion Recovery FLAIR sequence have been shown to characterise early glioblastoma infiltration and have been incorporated into post-operative planning of radiation therapy (4) and assessment of Glioma progression by Response Assessment in Neuro-oncology (RANO) criteria [16, 17]. However, precise anatomical localisation of tumour within these sequences is yet to be established. Newer functional imaging with positron emission tomography (PET) tracers, especially 18F-fluoroethyl-L-tyrosine (FET) PET [18–20], have shown some improvements in delineation of glioma but a highly sensitive and specific tracer is yet to be established [21].

Advanced MRI based tractography using diffusion tensor imaging (DTI) is sensitive in detecting the direction of water diffusion along white matter tracts. Studies have shown peri-tumoural changes in DTI that were not apparent in conventional MRI [22] and others have shown biopsies within abnormal white matter tracts seen on DTI have yielded active tumour [23]. Further to this, failure along white matter tracts has been associated with inferior outcomes [24], presumably due to a more infiltrative tumour biology.

Due to the uncertainties of tumour infiltration, most radiotherapy protocols use generous uniform or isotropic margins to account for microscopic tumour infiltration [25, 26]. However, this may lead to high doses of radiation to normal uninvolved brain tissue and contribute to long-term neurological toxicity such as cognitive, memory and functional abnormalities.

This study aims to characterise the failure pattern of treated temporal lobe (TL) glioblastoma on standard sequence MRI by comparing initial tumour location to the site of first failure, including potential infiltration along neural pathways.

## Methods

### Patient population

Adult patients with a new diagnosis of primary brain tumour referred to the Department of Radiation Oncology at the Northern Sydney Cancer Centre after May 2007 were entered into a prospective database, approved by the local Institutional Ethics Review Board. All patients with GBM involving the TL managed with Radiotherapy (RT) were retrospectively analysed.

Patients aged < 75 with good performance status received treatment consistent with the European Organisation for Research and Treatment of Cancer- National Cancer Institute of Canada (EORTC-NCIC) [1] Protocol, whilst elderly patients (aged > 75 years) or those with impaired Eastern Cooperative Oncology Group (ECOG) status were managed with hypofractionated RT (40Gy in 15 fractions) with or without systemic therapy. All patients were treated with Intensity Modulated Radiotherapy (IMRT) by one subspecialised radiation oncologist.

Patient, tumour and treatment factors were recorded into a prospective database. Performance status was assessed at the start of RT with the ECOG scale.

### Follow-up

All patients were followed closely with initial MRI at one month post RT (M + 1) then second monthly MRI until completion of adjuvant temozolomide or for six months, if not receiving chemotherapy, then three monthly until end of year 3 post RT, then four-six monthly until progression. Standard MRI sequences where obtained including T1/T2 weighted images pre and post contrast. T2 FLAIR sequences, Diffusion Weighted Imaging (DWI) and Apparent Diffusion Coefficient (ADC) sequences were also routinely acquired.

### Initial tumour location

**Fig. 1:**
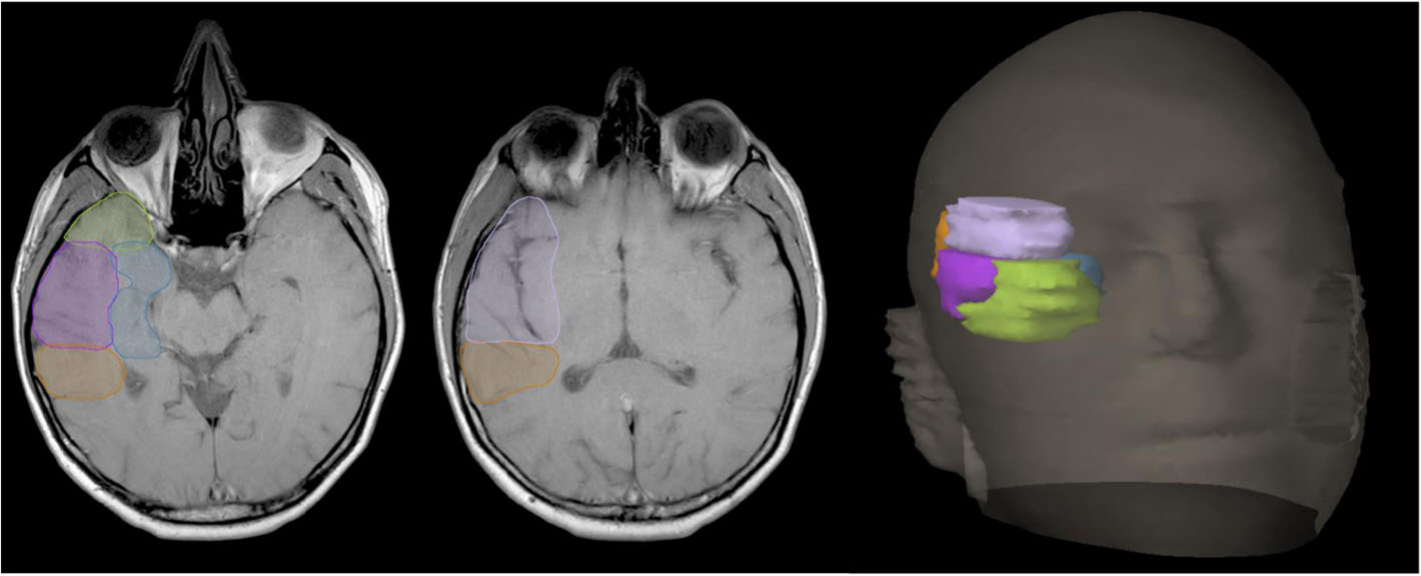
Local temporal lobe sites: anterior (green), lateral (purple), medial (blue), posterior (orange) and superior (lilac)

**Fig. 2:**
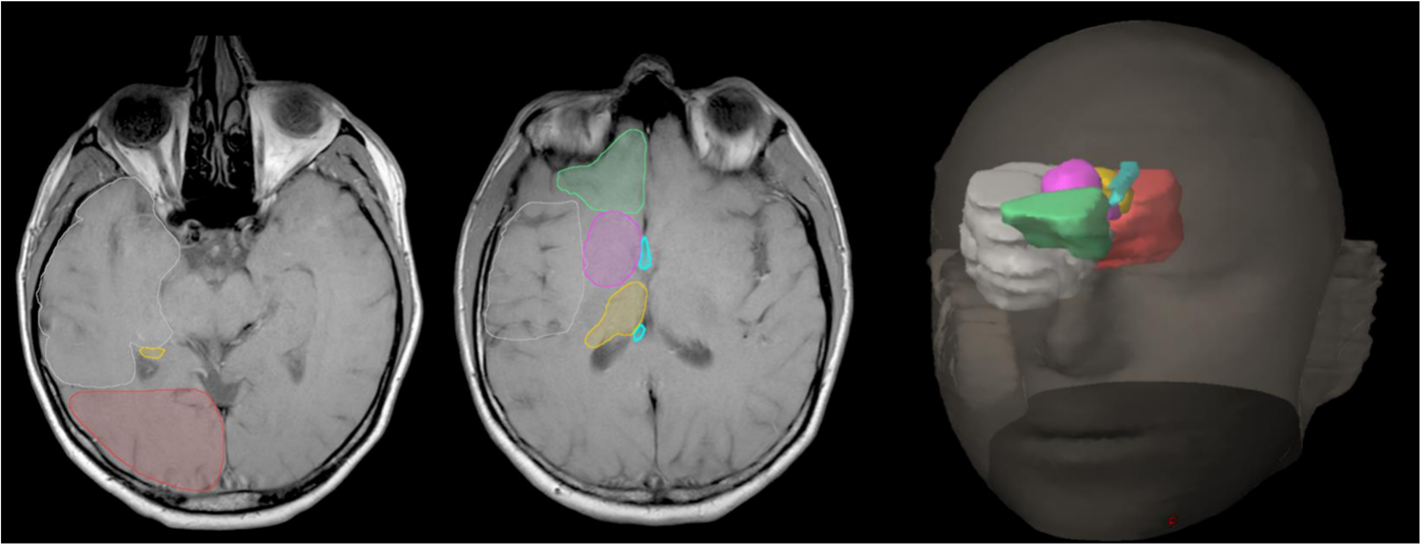
Five regional sites adjacent to temporal lobe (white): occipital lobe (red), inferior frontal lobe (green), caudate/internal/external capsules (pink), fornix (cyan) and thalamus/trigone of lateral ventricle (orange). Temporal lobe (white)

Site of initial tumour location at time of diagnosis was recorded and categorized into five TL sites; anterior, lateral, medial, posterior and superior (Fig. 1). Tumours involving regional sites past the temporal lobe were also categorized into 5 adjacent sites including the occipital lobe, inferior frontal lobe, caudate/internal/external capsules, fornix and thalamus/trigone of lateral ventricle (Fig. 2). Distant tumour involvement, where present, was classified into ventricular, contralateral hemisphere, brainstem, leptomeninges and spine. Tumours overlapping and involving multiple sites were coded for each site of involvement and analysed accordingly.

### Neural pathway involvement

**Fig. 3:**
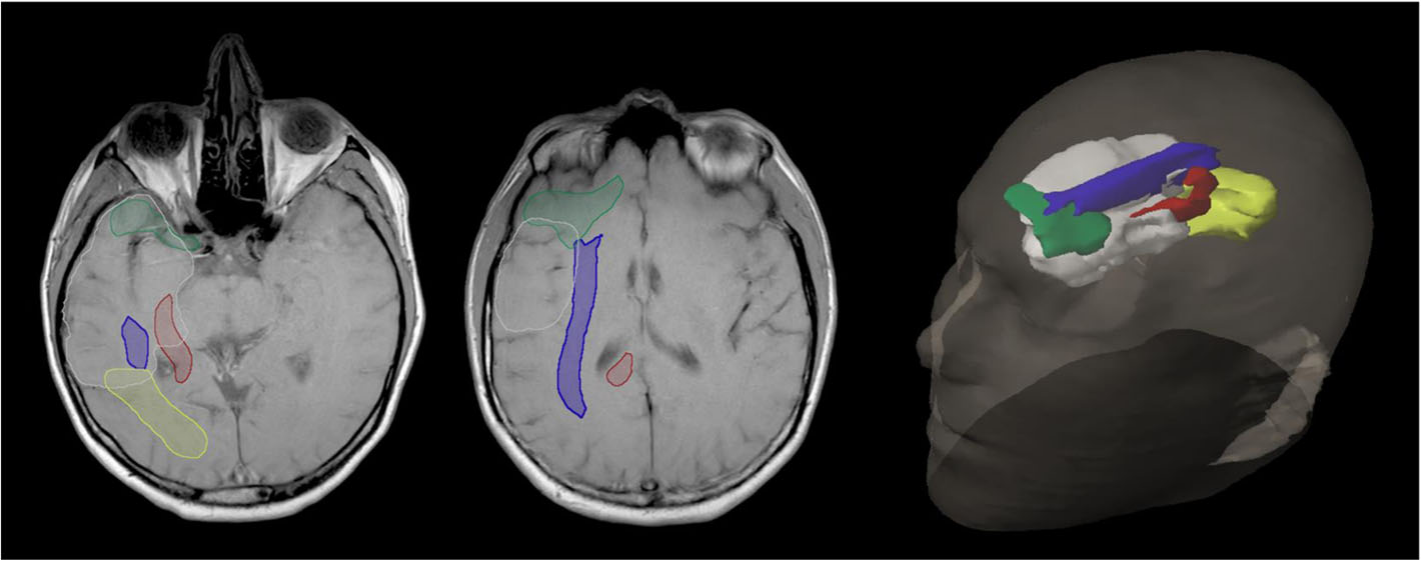
Four neural pathways adjacent to the temporal lobe (white): superior (green), anterior (blue), medial (red) and posterior (yellow)

Using established neural pathways atlases [27], potential neural pathway involvement was noted. Potential tumour involvement was classified by clinician discretion if within close approximation or seemingly tracking along neural pathways (Fig. 3). Specifically the pathways were superior (uncinate fasciculus), anterior (inferior longitudinal fasciculus), medial (fornix, cingulum) and posterior (inferior fronto-occipital fasciculus).

### Recurrent tumour

Recurrence was noted as the date of first MRI scan showing recurrent tumour or clinical deterioration not thought to be pseudoprogression. Recurrence was defined based on careful review of subsequent MRI from date of suspected MRI recurrence and any worsening clinical symptoms in line with RANO Working Group criteria [17]. All patients were discussed in a multidisciplinary tumour board meeting (MDT) with the involvement of treating physicians, diagnostic radiologists and nuclear medicine physicians. At time of recurrence, tumour location was noted and classified into local, regional, distant and pathway failure as per the above classifications.

### Statistical evaluation

Chi-Square test of independence was conducted on all patients to test if an association existed between the initial tumour location and the sites of first recurrence or neural pathway involvement. Pretreatment prognostic factors including age, extent of resection, Recursive partitioning analysis (RPA) Class and performance status were analysed with Kaplan-Meier survival analysis and log rank test to calculate the median survival for each sub-group and *p*-value to determine if any significant difference could be found. Cox regression model was used to analyse the impact of pretreatment prognostic factors on time to tumour recurrence. All reported *p* values were two-tailed. Statistical significance was defined as *p* ≤ 0.05. IBM SPSS Statistics Version 24 was used for statistical analysis.

## Results

335 consecutive GBM patients received IMRT between March 2007 and July 2014. 100 patients had tumours located within the TL. Of these, 86 had radiological progression and were included in the study. The median progression free survival was 7.1 months (95% CI 5.3–8.7) with an overall survival of 17.3 months (95% CI 13.8–18.1). The individual patient characteristics have been summarised in Table 1.

**Table 1:** Patient characteristics

Characteristics	Patients (Percentages)
Gender:	
Male	49 (57)
Female	37 (43)
Median Age:	63
Surgery:	
Biopsy	7 (8)
STR	32 (37)
GTR	47 (55)
Adjuvant treatment:	
Long course adjuvant RT/TMZ	65 (75)
Hypofractionated RT/TMZ	4 (5)
Hypofractionated RT	17 (20)
TMZ alone	0 (0)
Radiotherapy dose:	
60Gy	65 (76)
40Gy	21 (24)
Re-resection rates:	
No further craniotomy	70 (81)
Re-resection	16 (19)

GTR – Gross Tumour Resection

STR – Sub-Total Resection

TMZ – Temazolamide

### Initial tumour location

At initial diagnosis 81/86 (94%) patients had tumours confined to the TL only, with 3 (4%) patients having regional involvement (occipital lobe, fornix and trigone of lateral ventricle) and 2 (2%) patients having distant disease (ventricles). Of the patients with TL disease only, 60/81 (74%) had tumours localised to within one TL subsite. 16 (19%) had neural pathway disease at initial pre-treatment MRI.

### Tumour recurrence site

The majority of relapses involved the TL with 72 patients (84%) having a component of local failure, of which 22 (26%) had involvement of multiple TL sites. Involvement of regional sites (48%) and distant sites (26%) also occurred at initial relapse. 46 patients (53%) patients were noted to have neural pathway involvement at time of recurrence (Table 2). In regards to isolated relapses, 35 patients (41%) had relapse confined to the TL alone; 64 (74%) both local and regional sites; 3 (3%) in regional sites; 14 (16%) regional and distant sites and 8 (9%) were distant relapses only.

**Table 2:** Sites of recurrence of temporal lobe tumours

	Local recurrence:		Reg recurrence:		Distant recurrence:	Neural pathway recurrence:	
Anterior/Pole: *n* = 22	Anterior	Superior	Inferior Frontal Lobe		Ventricle	Superior	
	11 (50%)	10 (45%)	6 (27%)		3 (13%)	11 (50%)	
Lateral: *n* = 32	Lateral		Tail of Caudate		Contralateral Hemisphere	Superior	
	28 (87.5%)		11 (34%)		2 (6%)	6 (19%)	
Medial/Hippocampal: *n* = 15	Medial		Fornix	Occipital	Ventricle	Medial	Posterior
	15 (65%)		7 (30%)	6 (26%)	9 (39%)	10 (43%)	7 (30%)
Posterior: n = 15	Posterior		Occipital		Ventricle	Posterior	
	12 (80%)		8 (53%)		4 (27%)	8 (53%)	
Superior/Insular: *n* = 17	Superior		Inferior Frontal			Superior	
	15 (88%)		4 (24%)		0 (0%)	8 (47%)	

### Predictable sites of recurrence

**Fig. 4:**
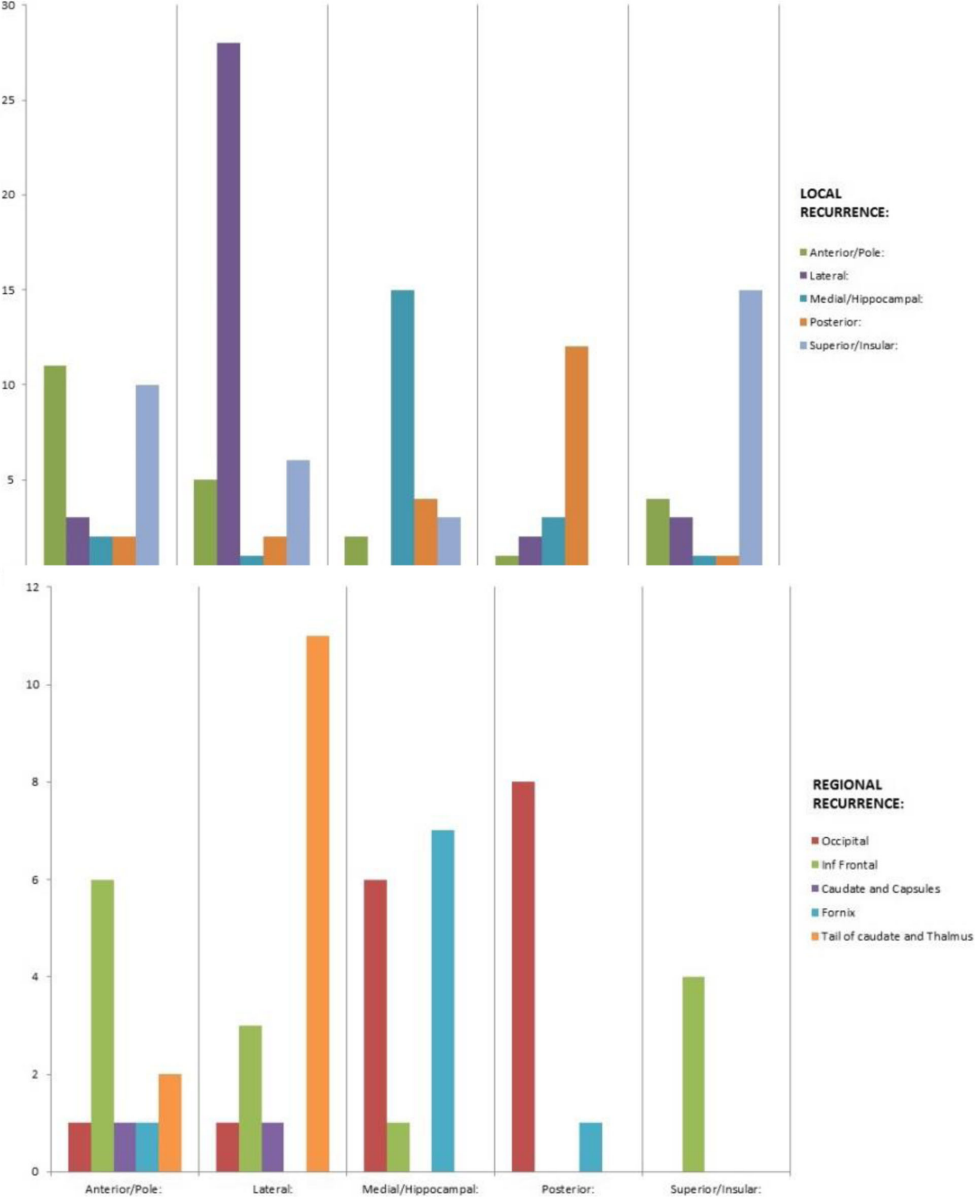
Local and Regional Patterns of Failure

**Fig. 5:**
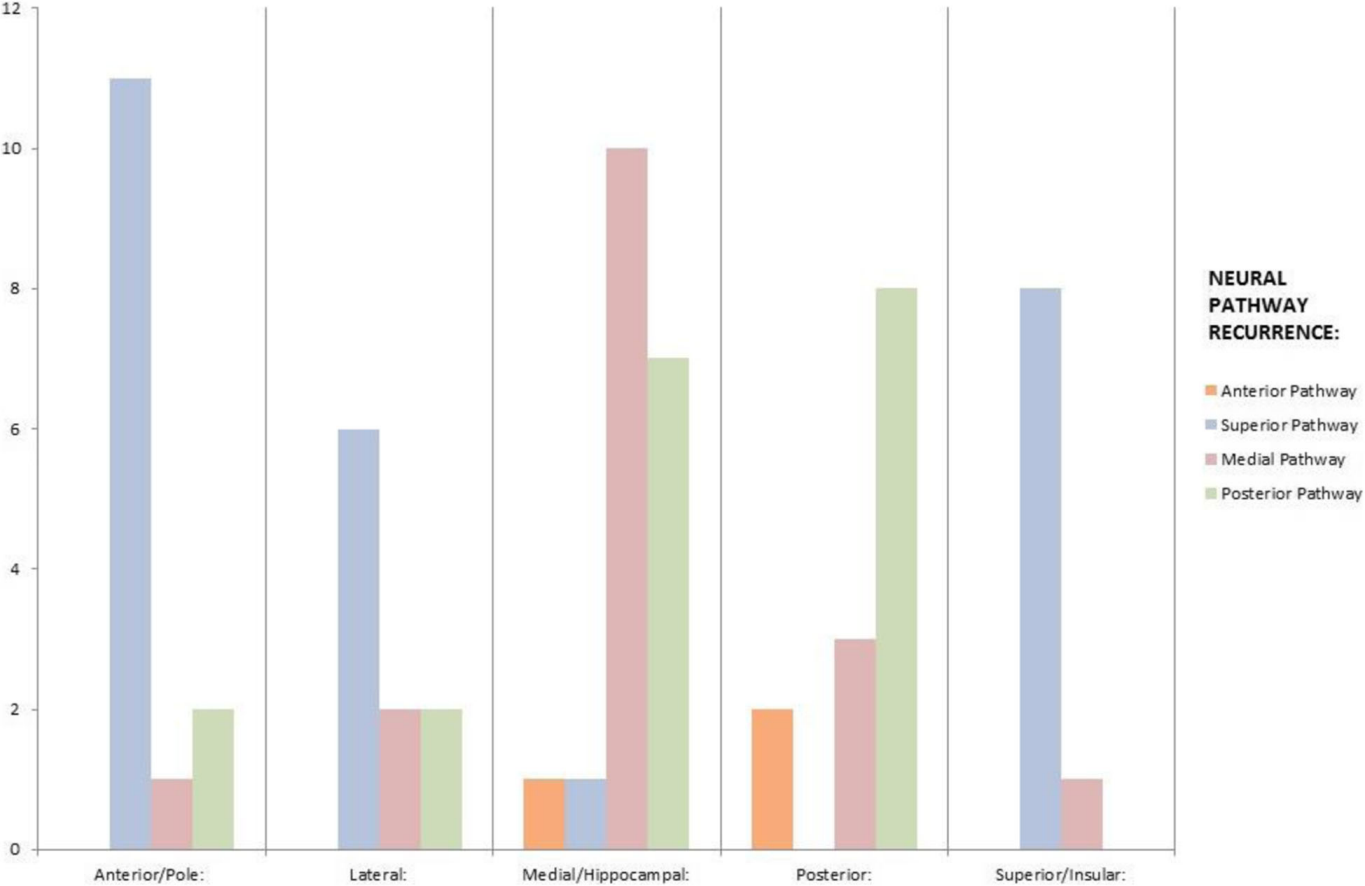
Neural Pathway Patterns of Failure

Initial TL site location predicted for local site recurrence (*p* < 0.0001), regional site recurrence (*p* = 0.004) and neural pathway recurrence pattern (*p* = 0.005), but not for distant sites (*p* = 0.081). Figure 4 shows distribution of local and regional recurrences dependent on initial origin of tumour within the TL and Fig. 5 shows neural pathway recurrences.

### Prognostic factors for relapse pattern

For the whole group of TL tumours, median survival was improved for patients managed with higher dose RT of 60Gy versus 40Gy with 17 months and 11 months respectively (*p* = 0.01, HR = 0.489, 95% CI = [0.274, 0.871]) but not with age (*p* = 0.17, HR = 1.486, 95% CI = [0.835, 2.646]), resection type (*p* = 0.24, HR = 0.751, 95% CI = [0.464, 1.218]), ECOG Performance Status (*p* = 0.11, HR = 1.617, 95% CI = [0.889, 2.942]), and RPA Class (*p* = 0.1, HR = 2.14, 95% CI = [0.851, 5.38]).

Assessment of local, regional and distant pattern of failure was undertaken with respect to prognostic factors mentioned above using cox regression modeling. On Univariate analysis, patients with sub-total resection (STR) or biopsy had a higher rate of regional failure (*p* = 0.002, HR =2.966, 95% CI = [1.468, 5.992]), but no other significant difference were seen between prognostic factors and local or distant failures. No statistical differences were seen on multivariate analysis.

The influence of extent of resection and failure site is described further in Table 3. Specifically, extent of resection did not predict for local failure with 86% of patients with gross total resection (GTR) failing at initial local site compared to 80% with subtotal resection or biopsy (*p* = 0.40). However those patients with subtotal resection or biopsy had a greater proportion of regional (63% vs 33%: *p* = 0.01) and distant failure (43% vs 18%: *p* = 0.01) than those with GTR. When regional failure occurred, it developed earlier in patients with subtotal resection than those with GTR (p = 0.01). Isolated regional or distant failure occurred in 14 patients and this was not influenced by extent of resection (*p* = 0.44).

**Table 3:** Patterns of Failure in relation to Extent of Resection

	Local Failure	Regional Failure	Distant Failure	Isolated Regional or Distant Failure
Subtotal Resection or Biopsy (*n* = 41)	80%	63%	43%	20%
Total or Near-total Resection (*n* = 45)	86%	33%	18%	14%

There was no significant difference between site of relapse and time of death from relapse. The median for the total group was 6 months, and there was no difference between failures at local, regional or distant sites (*p* = 0.65).

### Outcome for neural tract relapse

Initial neural pathway involvement was present in 16 patients (19%) and was associated with a subsequent greater rate of subtotal resection. At time of relapse, 46 patients (53%) were noted to have neural pathway involvement. For patients with subtotal resection there was a trend for subsequent earlier tract failure with a median time of 10 months compared to 19 months, which did not reach statistical significance (*p* = 0.06). There was no one neural pathway that was associated with the 14 patients with distant only failure (*p* = 0.34).

In regards time from neural tract involvement at failure and death, the median time to death was 7 months compared to 4 months in those without neural tract failure, and this did not reach statistical significance (*p* = 0.35).

## Discussion

Our results show that despite good initial tumour resection, with GTR and STR achieved in 55 and 37% respectively, 74% of patients had first documented failure isolated to local and adjacent brain sites. Although our study involved temporal lobe tumours only, others have shown similar rates of local failure elsewhere in the brain [10–12], suggesting that this is not unique to this anatomical location alone. Historically, this has been the case in the pre-temozolomide era, with dose escalations studies of up to 90Gy showing high infield failure rates of 89–91% [9, 28]. More recently, the use of temozolomide has become standard of practice since EORTC-NCIC protocol, especially in good performance status patients under the age of 70, showing an improvement in median survival of 14.6 months and an improvement in 5 year survival with concurrent and adjuvant temozolomide of 9.8% compared with 1.9% radiotherapy alone [29].

Despite these improvements in overall survival, modern series still show a failure rate of 70–85% local failure rate within 2-3 cm of the tumour cavity or directly within the high dose radiotherapy portals [9, 10, 28, 30, 31]. Some studies have reported a change in failure pattern in patients with GBM and 06-methylguanine DNA methyltransferase (MGMT) methylated tumours. Brandes et al. [31] reviewed 95 patients treated with high dose radiotherapy who showed an infield or marginal recurrence rate of 85% in unmethylated MGMT tumours, compared with 57.9% of patients with methylated tumours; with failures out of field presenting at significantly longer time intervals to those with in field failures. Therefore, knowledge of anatomical regions of failure and possible mechanisms of tumour spread may aid in improving tumour control in these patients.

There is uncertainty as to the optimal margins for adjuvant radiotherapy in GBM. The EORTC uses a single-phase technique in which the gross tumour volume (GTV) is defined as the surgical tumor bed plus any residual enhancing tumor. This is expanded by 2 cm to create the clinical target volume (CTV) and another 0.3–0.5 cm for the planning target volume (PTV) [32]. The Radiation Therapy Oncology Group (RTOG) protocols use a two phase technique with the first phase encompassing the primary contrast enhancing tumour and peri-tumoural oedema with a 2–2.5 cm expansion treated to a dose of 46Gy. The second phase is purely the contrast enhancing tumour with an expansion of 2–2.5 cm to an additional dose of 14Gy. However, the use of isotropic marginal expansions such as these may lead to overtreatment of normal brain tissue with no clear advantage in local control or survival. High dose radiotherapy to brain tissue has been shown to increase neurotoxic side effects including radionecrosis and cognitive decline [33, 34]. The volume of irradiated brain is believed to be associated with development of these complications [35], and treating smaller volumes should theoretically reduce these effects. As such, there is a need to improve tumour target delineation and differentiate between normal brain tissue as this may improve local control and reduce neural toxicity.

**Fig. 6:**
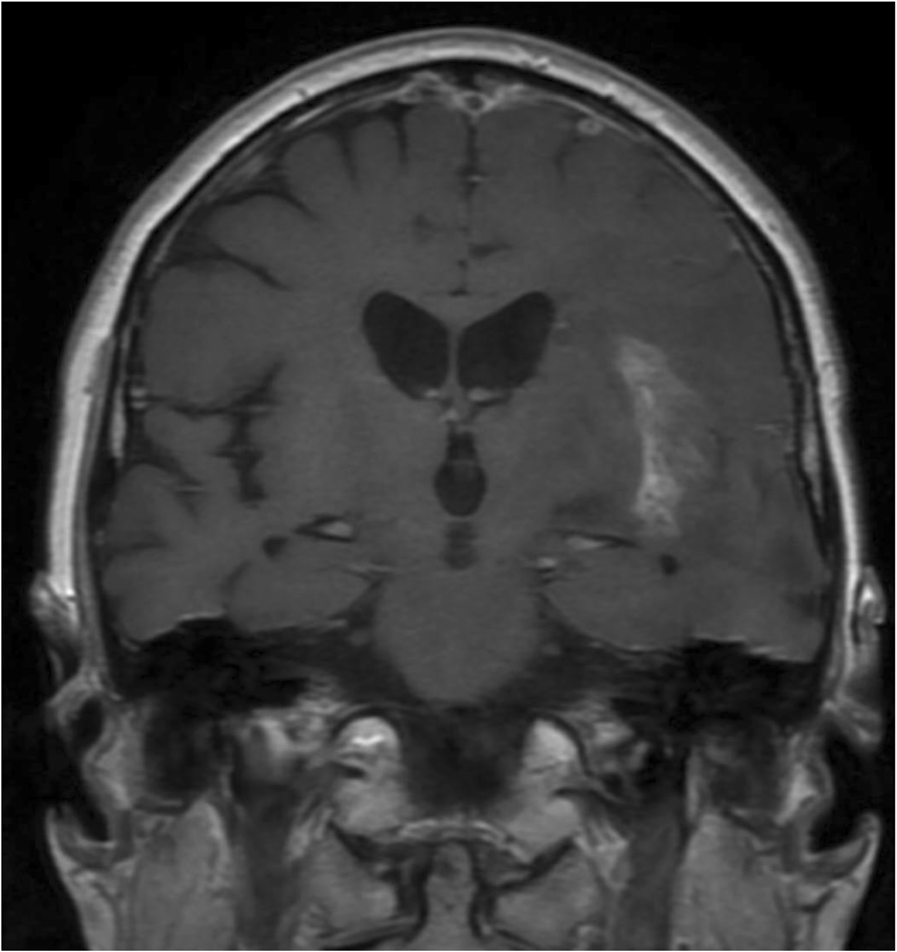
Temporal pole tumour recurrence: insular lobe involvement through the uncinated fasciculus

**Fig. 7:**
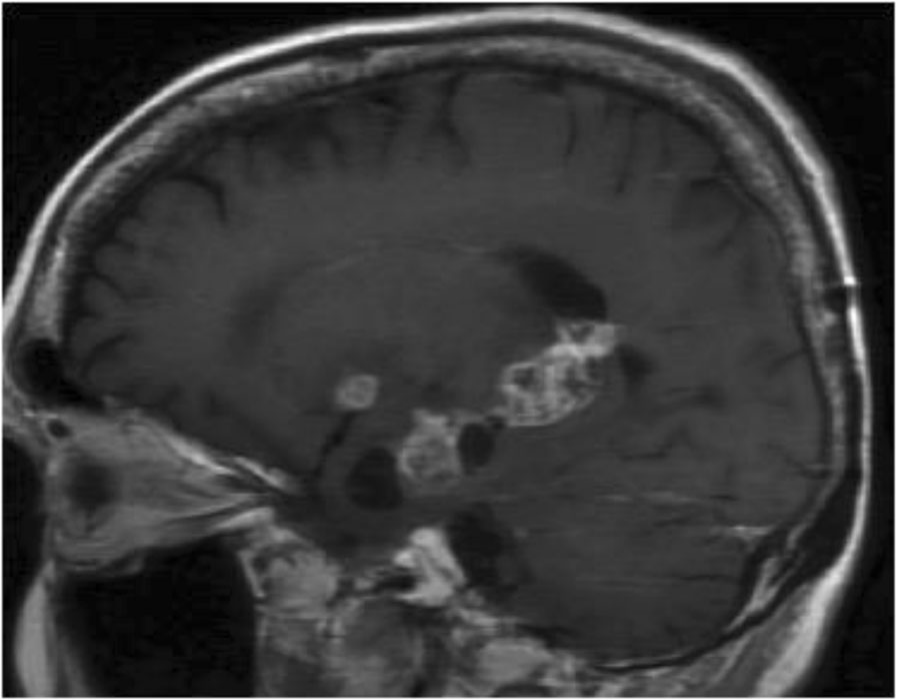
Posterior medial temporal lobe tumour recurrence: tumour infiltration of the splenium through the cingulum pathway

Whilst a component of local failure remains the main site of failure in the majority of patients, 15–20% of patients have an isolated regional or distant failure that appears to be related to tract involvement. Similarly patients with relatively sensitive tumours such as those with MGMT methylation, may have a proportionally greater rate of isolated tract associated regional or distant failure. This study showed 19% of TL tumours had neural pathway involvement at time of diagnosis and 53% of patients with neural pathway failures at time of first progression. Local (*p* < 0.0001), regional (*p* = 0.004) and neural pathway failures (*p* = 0.005) were predictable and depended on the initial location of tumour within the TL. For instance patients with temporal pole tumours predominantly relapse patterns extending into the insular and inferior frontal lobe, which is connected via the uncinate fasciculus (Fig. 6). Medial tumours extending into the hippocampus had involvement tracking into the roof of the third ventricle via the fornix; or if more posterior via the cingulum to the region of the splenium (Fig. 7).

Future strategies may involve the development of new radiation target volume delineation protocols incorporating potential microscopic disease along these predictable adjacent regions may impact on disease natural history in selected patients. Given modern modulated radiotherapy techniques, this could allow for selected dose escalation along sites of greatest risk of recurrence. In other patients who will have predominantly local in-field failure, the knowledge of relapse sites and non-isotropic margin expansion that concentrates on neural pathways may limit the amount of normal tissue treated. Ideally molecular or radiological research that may identify patients at greatest risk for relapse along neural pathways would assist further in the development of such protocols.

Others have evaluated neural pathway involvement by high grade gliomas at diagnosis and recurrence using advanced MRI techniques [23, 36]. Price et al., showed that DTI based MRI can improve tumour delineation and assess early infiltration along neural pathways with biopsies along involved neural tracts proving positive for tumour cells [23]. DTI has also been used to assist with surgical planning of tumour resections and more recently in voluming of radiotherapy portals [37, 38]. Berberat et al. used DTI based MRI planning in treating 13 patients with GBM, showing a total reduction in treated PTVs when compared to PTVs generated by conventional T2 weighted MRI [38]. Others have shown that tract involvement can also impact survival, especially major tract involvement such as the corticospinal and spinothalamic tracts [24].

The impact of relapse involving the neural pathways remains uncertain. In this patient cohort, salvage therapies were individualised not only based site of relapse and resectability, but also performance status at relapse, time from initial diagnosis and ability to tolerate or fund salvage systemic therapies such as Bevacizumab. The median survival from relapse to death was 6 months and this was not influenced by the pattern of relapse. Whether better coverage of neural pathways in patients whose tumours are sub-totally resected or involving neural pathways initially may delay relapse, or convert the pattern of relapse to more localised disease pattern, is uncertain. If knowledge of predictable sites of relapse is incorporated into protocols then at least significant normal tissue sites may be avoided in radiation portals, thus minimising the potential neural toxicity that may occur when combining radiation therapy with sensitising targeted therapies.

Limitations of this study include its retrospective nature with inherent problems of selection bias. Neuro-radiologists within the MDT judged tumour failure based on anatomical location and application of established neural pathway atlas information in approximating failure patterns, which may have also led to bias. Limited information was available on molecular features of the tumours analysed in this study. Only 16 patients had available results of MGMT promoter methylation status of which only 2 were positive. Another weakness involves the difficulty of distinguishing tumor progression from pseudoprogression, though we attempted to minimise the impact of this by considering subsequent changes in MRI scans and correlating this with clinical criteria for progression as per the RANO Working Group [17]. Furthermore, MRI was conducted at frequent intervals at our institution and usually at the same radiology practice which aided careful comparison and correlation with clinical findings. Additionally, our rates of locoregional and distant failure were similar to other published series. Further evaluation of neural pathway involvement could be done so in a prospective setting with functional imaging, such as DTI, with close correlation of local control and survival.

## Conclusions

Despite aggressive local treatment with maximal surgical resection and adjuvant radiotherapy, most GBMs fail locally or within the high dose radiotherapy portal. Despite much evaluation, there is no consensus on tumour delineation of GBM in radiotherapy. Neural pathway involvement by GBM has been noted by others, and this study supports the theory of neural pathway infiltration as a pattern of failure. This may be an important consideration for radiotherapy delineation of tumour volumes and avoidance of normal brain tissue and may be of value for prospective evaluation.

## Abbreviations

ADC: Apparent Diffusion Coefficient
CTV: Clinical Tumour Volume
DTI: Diffusion Tensor Imaging
DWI: Diffusion Weighted Imaging
ECOG: Eastern Cooperative Oncology Group
EORTC-NCIC: ᅟ
FLAIR: Fluid Attenuated Inversion Recovery
GBM: Glioblastoma Multiforme
GTR: Gross Tumour Resection
GTV: Gross Tumour Volume
HGG: High Grade Glioma
IMRT: Intensity Modulated Radiotherapy
MDT: Multidisciplinary Tumour Board Meeting
MGMT: 06-Methylguanine DNA Methyltransferase
MR: ᅟ
MRI: Magentic Resonance Imaging
OS: Overall Survival
PFS: Progression Free Survival
PoF: Patterns of Failure
PTV: Planned Tumour Volume
RANO: Response Assessment in Neuro-oncology
RPA: ᅟ
RT: Radiotherapy
RTOG: ᅟ
STR: Sub-Total Resection
TL: Temporal Lobe
